# X-Linked Lymphoproliferative Disease in Latvia: A Report of Two Clinically Distinct Cases

**DOI:** 10.1155/2020/7108657

**Published:** 2020-07-21

**Authors:** Ieva Nokalna, Madara Kreile, Dagnija Butane, Zhanna Kovalova, Zanda Daneberga, Edvins Miklasevics, Dace Gardovska, Linda Gailite

**Affiliations:** ^1^Riga Stradins University, Riga, Latvia; ^2^Childen's Clinical University Hospital, Riga, Latvia

## Abstract

X-linked lymphoproliferative disease (XLP) is a rare primary immunodeficiency. Affected individuals usually present with the Epstein–Barr virus infection and have no apparent disease prior to presentation. The most common clinical manifestations are fulminant infectious mononucleosis, dysgammaglobulinaemia, and lymphoma (usually of B-cell origin). XLP is caused by mutations in the *SH2D1A* gene which encodes the intracellular adaptor molecule SAP (signalling lymphocyte activation molecule- (SLAM-) associated protein). SAP is predominantly expressed in T cells and NK cells and functions to regulate signal transduction pathways downstream of the SLAM family of surface receptors to control CD4+ T cell (and by extension B-cell), CD8+ T cell and NK cell function, and development of NKT cells. Thus, SAP mutations cause dysregulation of the immune system, with defects in both cellular and humoral immunity. Here we report two clinical cases of three patients who presented with different manifestations of XLP, namely, fulminant infectious mononucleosis, Burkitt lymphoma and hypogammaglobulinaemia.

## 1. Introduction

X-linked lymphoproliferative disease (XLP) is a rare primary immunodeficiency. It is caused by loss-of-function mutations in the *SH2D1A* gene which encodes the intracellular adaptor molecule SAP (signalling lymphocyte activation molecule- (SLAM-) associated protein) [1]. These cause immune dysregulation usually in response to the Epstein–Barr virus (EBV) or other unidentified antigenic stimuli or due to ineffective T and NK cell interactions with B cells. There is dysfunction of both cellular immunity (cytotoxic T lymphocyte and NK cell dysfunction resulting in decreased cytokine production) and humoral immunity (abnormal immunoglobulin levels and abnormal antibody responses to infections and vaccinations) in XLP.

The most common clinical manifestations of XLP are haemophagocytic lymphohistiocytosis (HLH), dysgammaglobulinaemia, and lymphoma; however, aplastic anaemia, vasculitis, chronic gastritis, and skin lesions have also been described [2–4]. Despite their underlying immunologic abnormalities, the majority of patients have no symptoms prior to presentation usually with EBV infection which triggers the clinical manifestations, perhaps because of the B-cell tropism of this virus. After infection, EBV remains latent in B cells for the lifetime of the patient, and although most people remain asymptomatic, it can cause B-cell and T-cell lymphomas, Hodgkin lymphoma, and Burkitt lymphoma in certain groups, particularly in immunocompromised patients [2, 5]. It could be that exposure of XLP patients to EBV induces an uncontrolled immune response involving activated monocytes and lymphocytes. Despite this immune activation, XLP patients fail to control EBV infection, resulting in fulminant infectious mononucleosis. However, patients can also develop hypogammaglobulinaemia and B-cell lymphoma independently of exposure to EBV. Thus, XLP must be thought of as a disorder of immune dysregulation and not solely as a condition triggered by EBV [1, 2].

The spectrum of phenotypes seen in XLP is varied. HLH is the main and lethal presentation, usually occurring in early childhood, and associated with significant mortality. It is a multisystem syndrome caused by hyperinflammation resulting in immune dysregulation, tissue damage, and often multiorgan failure. The main features are fever, cytopenias, and hepatosplenomegaly. Additionally, the involvement of other organs is frequently seen [2, 5]. Lymphomas (primarily non-Hodgkin type, B-cell lymphomas) occur in approximately one-third of patients with XLP. The excessive proliferation of B cells may be due to impaired immune surveillance and elimination by T cells [4, 6]. Dysgammaglobulinaemia can also be seen in approximately one-third of XLP-affected males and has the best prognosis, especially when treated with immunglobulin replacement therapy. Some patients with dysgammaglobulinaemia secondary to XLP can be misdiagnosed with common variable immunodeficiency (CVID) [6–8].

Management of XLP consists of treatment of disease manifestations, preventative therapy, and curative therapy. For the treatment of acute EBV infection immune globulins, antiviral agents can be used. For prevention of further sequelae, immune globulin replacement therapy has been used [9], and an alternative for both treatment of disease manifestations and preventative therapy is ablative B-cell therapy with rituximab (anti-CD20), that has shown to control acute primary EBV infection, prevent severe EBV infection, and clinical deterioration prior to allogeneic hematopoietic cell transplantation (HCT) in EBV-negative patients [10]. In the case of development of HLH, patients should be promptly treated with HLH-specific therapy to induce remission prior to HCT. HCT is currently the only definitive treatment for XLP, that should be recommended in both symptomatic and asymptomatic XLP patients, in order to improve the final outcome [11].

There are no well-defined correlations between genotypes and clinical phenotypes of XLP. Genetic analyses have detected deletions and splice site and nonsense and missense mutations in *SH2D1A*, but to date, clear correlations between pathogenic variants/mutations and severity of phenotypes have not been established. Here, we report two different presentations of *SH2D1A* mutations. Written informed consent was obtained from the parents of each patient.

## 2. Case 1

This is the index case of a previously healthy 13-month-old male infant born in a nonconsanguineous Caucasian family. The child was admitted to hospital because of a fever and rash; the parents attributed the rash to the anti-inflammatory drugs administered to reduce the boy's temperature. The boy was born at term with a weight of 3,470 g and a height of 51 cm. At the time of admission, the patient's general condition was fair and his initial diagnosis was acute viral respiratory infection and rash of unknown aetiology, probably allergic. Blood analysis did not reveal any remarkable changes in total blood cell count or biochemical parameters.

During the first few days of hospitalization, the child had a moderate fever, diarrhoea, and maculopapular rash. On the fourth day of hospitalization, as the child's general condition had not improved, antibacterial drugs were administered; however, no significant improvement was observed. On the seventh day of hospitalization, the child's health suddenly deteriorated. He exhibited a high degree of fever, his rash was progressing, and he had hepatosplenomegaly. Laboratory analyses revealed the following: thrombocytopenia 34,000 (reference range 229,000–553,000); total leukocyte count was 8.26 × 10^6^, 84% were lymphocytes; moderately increased liver transaminases Alt 225 U/L (reference range < 33 U/L), and Ast 520 U/L (reference range < 33 U/L); and C reactive protein was 34.26 mg/dL (reference range 0–5 mg/dL); coagulation tests revealed decreased clotting. Polyserositis was diagnosed by ultrasound investigation. Extra fluid and a therapeutic dosage of prednisolone were introduced, resulting in stabilization of the child's condition. After two days, the child's condition started to deteriorate again; he was lethargic, he did not display diuresis, and his feet were oedematous. His glucose level was 0.6 mmol/L (reference range 2.8–5.4 mmol/L), Alt 2,213 U/L, and Ast 2,989 U/L. At this point, he was transferred to the intensive care unit where a consulting haematologist suspected EBV infection, which was subsequently confirmed serologically. A bone marrow aspirate sample was collected for diagnostic purposes, and severe dishaematopoiesis without any traces of haematopoiesis and signs of macrophage activation syndrome was found. A final diagnosis of HLH was made ex consilio based on the clinical picture and laboratory analyses (thrombocytopenia, anaemia, neutropenia, decreased clotting, hypertriglyceridaemia, hyperimmunoglobulinaemia M, liver cytolysis, increased cell count (liquid portion), hyperferritinaemia, and hypoproteinaemia). Therapy comprising dexamethasone, etoposide, cyclosporine, and methotrexate was prescribed.

Two weeks after hospitalization, the child died from multiple organ failure. The autopsy reported haemorrhagic changes in the lungs, liver, spleen, and gastrointestinal tract, subtotal liver necrosis, kidney necrosis, ascites, and total bone marrow suppression.

One and a half years later, the deceased child's previously healthy 13-month-old brother was hospitalized with a low-grade temperature and rash covering his legs. The boy was born at 40/41 weeks with a weight of 3,470 g and a height of 52 cm; the mother's pregnancy was uneventful. During the hospitalization of their first child, it was noted that the parents had limited education and did not always fully understand the situation after several explanations; however, it was viewed that they provided adequate care for their children.

Over the next few days, the child slowly deteriorated; he had a febrile temperature and his rash progressed. On the third day of hospitalization, the child was transferred to the intensive care unit due to massive oedema, hepatosplenomegaly, thrombocytopenia 69,000, hypoproteinaemia, elevated levels of liver transaminases, and decreased clotting. He was found to have EBV infection. Based on the clinical picture, laboratory analyses, and medical history of his deceased brother, HLH treatment was initiated, although due to severe liver injury, his prognosis was poor. After 12 days of hospitalization and following unsuccessful cardiopulmonary resuscitation, the patient died. As XLP was suspected, DNA was isolated from both siblings and their mother. Immunoglobulin levels and lymphocyte subsets of second brother at time of hospitalization are shown in Table 1.

The Sanger sequencing analysis for the coding exons of the *SH2D1A* gene was conducted. For both of the siblings, it was not possible to amplify exons 1, 2, and 3 of the *SH2D1A* gene; exon 4 generated an amplification product, but no pathogenic variants were detected. In addition, integrity and quality of the extracted DNA samples were tested and evaluated as sufficient for the sequencing analysis. This suggested that the inability to amplify exons 1, 2, and 3 may have arisen from a large deletion within the *SH2D1A* gene. The possible presence of the large deletion within the *SH2D1A* was suggested but not confirmed.

In order to verify large rearrangements within the *SH2D1A* gene, we performed a genomewide analysis of genomic variants with the arraySNP HumanCytoSNP-12 v2.1 BeadChip Kit (Illumina) according to the manufacturer's instructions. Data analysis was performed with the BlueFuse Multi v4.4 and Genome Studio (with CNV Partition plug-in) software (Illumina). DNA samples of the two patients and their mother were evaluated for the presence of a large deletion within the *SH2D1A* gene. The patients' microarray analysis revealed a male hybridization pattern with approximately 192,000 bp deletion of chromosome X at band q25: arr [GRCh37]Xq25(123479999_123672135)x0. The lost region contained the *SH2D1A* gene and part of the *STAG2* and *TENM1* gene (DECIPHER). To confirm precise deletion breakpoints, primers corresponding to the breakpoint regions were designed and a 261 bp fragment containing breakpoint was amplified from the aberrant X chromosome by PCR. Sanger sequencing of this amplicon confirmed deletion of 197,730 bp. The maternal DNA sample revealed the same deletion found in the patients' DNA samples.

## 3. Case 2

**Figure 1:**
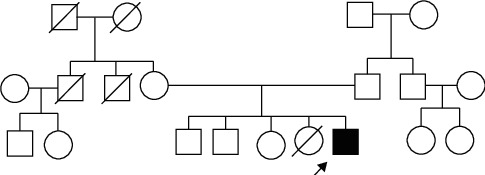
Family tree of the patient of the clinical case no. 2.

The second case is a 7-year-old boy with right-sided submandibular lymphadenopathy who was transferred from a regional hospital to the Children's University Hospital where he remained for approximately two weeks. His body temperature during this period was normal; however, his parents reported that he had occasional fatigue and poor appetite. It was noted that he had signs of dysmorphism and mild growth delay. He was born at 40 weeks (mother's 5th pregnancy, 5th birth) with a weight of 3,680 g and a height of 53 cm; there were no complications during pregnancy/birth. He had had some mild viral infections after starting kindergarten and had received all his immunizations according to the immunization schedule. His parents did not detail any chronic diseases. He had two healthy brothers, one sister with a double kidney, and one sister that had died at 11 years of age due to some type of congenital pathology, possibly microcephaly (not enough information was available)—family tree shown in Figure 1.

Ultrasound examination of the boy's cervical lymph nodes and abdominal cavity, thoracic CT scan, and abdominal MRI were performed, and they showed suspicions of lymphoproliferative disease. Consequently, lymph node biopsy, bone marrow aspiration, and trephine biopsy were performed; however, no evidence of lymphoproliferative disease was found, and the boy was discharged in a stable condition. At a follow-up visit, two months later, it was observed that the lymph node conglomerate in his neck area had increased in size. An MRI of the head and neck area was subsequently performed, and the following was reported: large abnormal formation in the cervical soft tissues on the right side, with extracranial parapharyngeal, carotid space with internal carotid artery (ACI), ACE overgrowth, intracranial proliferation through the foramen jugulare in the cerebral cortex with peripheral oedema, and 4th ventricle compression with initial 3rd and lateral ventricle extension. Trepanation, extirpation, and biopsy of the mass were performed, and high-grade B-cell lymphoproliferation with Burkitt lymphoma-specific morphology and immunophenotype was confirmed. Chemotherapy was initiated according to B-NHL-2004 protocol for the R3 treatment group (Burkitt lymphoma IV, CNS +) with prophase + chemotherapy blocks. During chemotherapy, the boy had several very serious episodes of infection with sepsis and bilateral polysegmental lung damage. He was also found to be EBV DNA and IgG and IgM positive.

After chemotherapy, the boy reached complete remission. However, persistent hypogammaglobulinaemia was noted (shown in Table 2), and consequently intravenous immunoglobulin was started.

As primary immunodeficiency was suspected, the boy was consulted by a geneticist. The Sanger sequencing analysis for the coding exons of the *SH2D1A* gene (NM_002351.5) was conducted. A hemizygous mutation was detected in the *SH2D1A* gene, NM_002351.4:c.5A > G, p.Asp2Gly, rs1556619319. It was classified as likely pathogenic, thus confirming a diagnosis of XLP. The classification was based on ACMG guidelines, fulfilling criteria PM1 (variant located in the functional domain); PM2 (variant not found in gnomAD exomes or genomes); PP2 (missense variant is the pathogenic mechanism in the *SH2D1A* gene); PP3 (multiple in silico tools suggesting pathogenicity); PP5 (reputable source, ClinVar classifies the variant as likely pathogenic) [12]. At present, the boy receives intravenous immunoglobulin regularly, and his condition is stable. Genetic testing for other family members were not performed.

## 4. Discussion and Conclusions

XLP is a rare primary immunodeficiency. The spectrum of phenotypes seen in XLP is varied, making the diagnosis of the condition very difficult [2]. These two case reports detail XLP patients with very different clinical presentations and outcomes. The two patients from the same family in Case 1 presented with EBV-associated HLH, while the patient in Case 2 presented with much milder clinical symptoms. We have evidence that he was exposed to EBV, but he did not develop HLH. His first manifestations were lymphoma and dysgammaglobulinaemia, which can initially be misdiagnosed as manifestations of CVID or secondary dysgammaglobulinaemia after chemotherapy.

There is evidence of some XLP patients with relatively mild clinical features, such as isolated dysgammaglobulinaemia, or who have survived EBV infection [2, 5, 13]. A possible explanation for these cases could be somatic reversion, i.e., a spontaneous genetic change in a disease-causing mutation at the level of a single lymphoid precursor cell that reverts the gene back to normal. Furthermore, certain mutations may result in differential lineage-specific regulation of *SH2D1A* expression, about which, little is known [14].

It has been postulated that mutations that remove or truncate the *SH2D1A*-encoded protein are more often associated with a severe phenotype, whereas missense mutations occur preferentially in mildly affected patients [5]. To our knowledge, this is the first time a phenotype of the missense mutation Asp2Gly has been described. It has been reported that approximately 25% of XLP patients have a deletion of one or more exons of the *SH2D1A* gene or are lacking the entire *SH2D1A* gene [15]. Mejstrikova et al. described an interesting case where the deletion of all the exons might be associated with skin lesions [16]. As the deletion detailed in Case 1 also includes part of the *TENM1* gene, the mother's intellectual deficit could be a consequence of the deletion in this gene, as previously reported [17]. However, to our knowledge, there are no reported cases of deletions in both genes, making the aforementioned proposal purely speculative.

Although HLH is the most common clinical presentation of XLP, lymphomas (primarily non-Hodgkin type, B-cell lymphomas) and dysgammaglobulinaemia occur in approximately one-third of XLP patients [4, 6]. Therefore, a diagnosis of XLP should be considered for all male patients with CVID or another hypogammaglobulinaemic condition, HLH (especially if associated with EBV infection and/or early mortality), severe infectious mononucleosis, or lymphoma (especially B-cell, non-Hodgkin lymphoma affecting extra nodal sites) [18]. Additionally, males with a known or even suspected family history of XLP should be tested for the disease to improve diagnosis and create a more efficient approach. Early genetic testing could have led to early diagnosis for the second brother, that would be an indication for hematopoietic cell transplantation, which could have been a life-saving intervention.

## Figures and Tables

**Table 1:** Immunoglobulin levels and lymphocyte subsets of the second brother.

*Immunoglobulin levels*	
IgG (*N* = 4.98–12.5 g/l)	1.2↓
IgA (*N* = 0.35–2.05 g/l)	0.81
IgM (*N* = 0.47–1.78 g/l)	3.7↑

*Lymphocyte subsets*	
CD3+ (%) (*N* = 55–78)	62
CD3+ CD4+ (%) (*N* = 27–53)	24↓
CD3+CD8+ (%) (*N* = 19–34)	35↑
CD19 (%) (*N* = 10–31)	20
CD16/56 (%) (*N* = 4–26)	16
CD4/CD8 (0.9–2.6)	0.77↓

**Table 2:** Immunoglobulin levels and lymphocyte subsets of patient No. 2.

*Immunogloulin levels*		Normal range
IgG (g/l)	1.6↓	4.98–12.5
IgA (g/l)	0.1↓	0.35–2.05
IgM (g/l)	0.95↓	0.47–1.78

*Lymphocyte subsets*		
CD3+ (%)	82.91↑	55–78
CD3+ (cells/*μ*l)	2.60	0.7–4.2
CD3+CD4+ (%)	35.25	27–53
CD3+CD4+ (cells/*μ*l)	1.10	0.3–2
CD3+CD8+ (%)	46.03↑	19–34
CD3+CD8+ (cells/*μ*l)	1.44	0.3–1.8
CD19 (%)	10.25	10–31
CD19 (cells *μ*l)	0.32	0.2–1.6
CD16/56 (%)	6.45	4–26
CD16/56 (cells/*μ*l)	0.2	0.09–0.9
CD4/CD8	0.77↓	0.9–2.6
WBC (×10^3^ *μ*l)	7.0	
Lymphocytes (%)	44.8	
